# The Coming Meeting of the Texas State Medical Association

**Published:** 1887-02

**Authors:** 


### EDITORIAL DEPARTMENT.

F. E. DANIEL, M. D., EDITOR AND PUBLISHER.

COLLABORATORS:

*E. J. Doering, M. D., Chicago.*
*Geo. Cupples, M. D., San Antonio.*
*Odo Betz, M. D., Germany.*
*E. J. Beall, M. D., Fort Worth, Texas.*
*T. C. Osborn, M. D., Cleburne, Texas.*
*Wm. Penny, M. D., New York.*
*H. O. Marcy, M. D., Boston.*
*C. K. Gregg, M. D., Mexico.*
*R. M. Swearingen, M. D., Austin.*
*C. H. Wilkinson, M. D., Galveston.*
*J. W. McLaughlin, M. D., Austin.*
*R. H. L. Bibb, M. D., Mexico.*

### THE COMING MEETING OF THE TEXAS STATE MEDICAL ASSOCIATION.

This important event will take place in Austin, on the 4th Tuesday in April '87, (26th day) and will continue four days.

The annual meeting of the Association marks an era in the life of the members; it is always looked forward to with interest, by them; but it is believed the coming meeting will be more attractive than any heretofore held. The roll now numbers some 500 members; eighty-nine, having joined at Dallas. Austin being the Capital, famed for its beauty and healthfulness, no less than for the liberal, open-handed hospitality of the citizens, is always attractive; and in the lovely month of April—the month of buds and breezes and bright flowers, it is just charming! Here will be assembled not less than six hundred of the leading physicians of the State; and perhaps some of the medical celebrities from other States will honor the occasion by their presence. The exhibit, too, of Pharmaceuticals and chemicals by the great manufacturers of the North and East, and of surgical instruments and appliances, it is expected, will be the most extensive and imposing of any yet made on similar occasions. Hence, it is believed that many physicians who have not heretofore attended the Association's meetings, will take advantage of this splendid opportunity—with reduced rates of travel—to visit the capital, it being central and easy of access from all points, and see for themselves; see the grand pile of native granite, which, under apparently magic influence, is so rapidly taking shape, and which, by that time, will be approaching completion. Many will doubtless bring their wives and daughters, and others, friends, for the same reasons. Certainly it will be a grand time, and no doubt our citizens will aid the committee of arrangement liberally, in preparing for our distinguished guests a reception worthy of them, and of the occasion.

It is impossible to handle so large a body in the manner of conducting a smaller one. The Association has really grown so great that it can no longer be made to conform to a programme cut out for it in its youth. We pointed out this fact after the Dallas meeting. We call attention to it now, and suggest to the committee of arrangements to provide three halls for the Section meetings, in addition to that for the general sessions, and those for exhibits, council meetings, &c. We suggest to class the Sections in three divisions, and devote an afternoon to each division; for it is impossible, now, to give an afternoon to each Section. It was attempted at Dallas, and the result was, some of the Sections were not reached at all, and the *majority* of the papers were not read—some not even read by title! There is nothing in the constitution and by-laws which forbids this arrangement; the latter simply says:

"The general meeting of the Association shall be restricted to the morning sessions; and the afternoons sessions, commencing at 3 o'clock, shall be devoted to the hearing of reports and papers, and their consideration, in the following Sections:" [Here the Sections are enumerated.]

It is suggested then, to have three Sections going at once, in three separate halls, after the manner of the American Medical Association. The time has come when we must double up, if we would get through with the work.

An important change we propose in the by-laws is, to allow physicians to join by direct application, as is done by the A. M. A. It will aid rapidly in bringing into the parent-fold all the desirable men in the profession in the State. At present, one can only become a member by being present, or by sending in his application *during* a *meeting*, accompanied by his diploma. We suggest that some active and zealous member make a memorandum *now*, and at the meeting propose such an amendment, or ordinance, as will enable a physician to become a member by making application to the President and Secretary, accompanied by a certificate from the Clerk of the District Court, under seal of the Court, that the applicant has a diploma from such and such a college, duly registered in that office. Let the application be indorsed by two members in good standing, and sent to the Secretary, together with the initiation fee and yearly dues. Or, if he is a member in good standing of a county society, let him furnish, in addition to the clerk's certificate, one from the Secretary of said Society. The advantage of this amendment is quite evident.

There are other changes of less importance which are, in our judgment, desirable, but of those, more anon.

The JOURNAL here and now, extends to the brethren the privileges of the *sanctum*, and invites them to make it headquarters, as it is of our local profession, and club-room of the County Medical Society. Here will be found all the latest medical literature—the leading medical journals and the latest books, together with a cordial weleome.

